# A Lesson Learnt from Food Chemistry—Elevated Temperature Triggers the Antioxidant Action of Two Edible Isothiocyanates: Erucin and Sulforaphane

**DOI:** 10.3390/antiox9111090

**Published:** 2020-11-06

**Authors:** Jakub Cedrowski, Kajetan Dąbrowa, Agnieszka Krogul-Sobczak, Grzegorz Litwinienko

**Affiliations:** 1 Faculty of Chemistry, University of Warsaw, Pasteura 1, 02-093 Warsaw, Poland; jcedrowski@chem.uw.edu.pl (J.C.); akrogul@chem.uw.edu.pl (A.K.-S.); 2 Institute of Organic Chemistry, Polish Academy of Sciences, Kasprzaka 44/52, 01-224 Warsaw, Poland; kdabrowa@icho.edu.pl

**Keywords:** isothiocyanates, sulforaphane, erucin, antioxidants, radicals

## Abstract

In this communication we demonstrate that two natural isothiocyanates, sulforaphane (SFN) and erucin (ERN), inhibit autoxidation of lipids at 140 °C but not below 100 °C. This effect is due to thermal decomposition of ERN and SFN to sulfenic acids and methylsulfinyl radicals, species able to trap lipidperoxyl radicals. Our observations shed new light on thermal processing of vegetables containing these two isothiocyanates.

Isothiocyanates (ITCs) can be found in vegetables in the form of glucosinolates (β-thioglucoside-N-hydroxysulfates), and during cutting, slicing, or grinding, ITCs are enzymatically evolved to give a gentle, specific flavor and taste. Recently, great attention has been paid to naturally-occurring ITCs with methylsulfoxy- and methylthio-moieties (see Figure 1), namely sulforaphane (4-methylsulfinylbutyl isothiocyanate, SFN) and erucin (4-methylthiobutyl isothiocyanate, ERN) [1,2,3,4,5].

Both ITCs are the components of our diet when consuming common *Cruciferous* vegetables [1,6]: SFN is present in broccoli, some cultivars of cabbage, and Brussels sprouts while ERN predominantly could be obtained from rocket [1,6,7,8]. Certain amounts of SFN along with ERN, could be provided from cabbage and red cabbage, rutabaga or turnip [6].

Talalay and co-workers [2,3,7] discovered and documented that among natural ITCs sulforaphane is the most potent inducer of carcinogen-detoxifying enzymes (e.g., quinone reductase, glutathione-S-transferase, heme oxygenase), and SFN is considered as one of the most potent chemopreventive phytochemical [2,7,9]. Such activity of SFN is due to induction of *Phase 2* enzymes of the xenobiotic metabolism (this process involves Nrf2 signaling) and inhibition of *Phase 1* enzymes. SFN and ERN are also recognized as inducers of some apoptotic pathways, anti-inflammatory effects, inhibition of angiogenesis, and their H_2_S-donating ability might be important in redox homeostasis agents [2,4,10,11,12,13,14]. ERN, a less studied analogue of SFN, also has a considerable impact on human health, being a potential chemopreventive and anticancer agent [3,8,12,14].

Impressive health benefits of ITCs have been connected with their ability to modulate cellular redox status due to induction of *Phase 2* cytoprotective enzymes [15] and such an effect has been named “the indirect antioxidant action” because there is no direct scavenging of radicals by ITCs [16]. The attempts to demonstrate that ITCs are able to effectively react with oxygen-centered radicals like peroxyls were unsuccessful (although glucoerucin, the parent glucosinolate of ERN, efficiently decomposes hydrogen peroxide and *tert*-butylperoxide proving that glucoerucin is a preventive antioxidant [17]). There is no experimental evidence that ITCs can inhibit or retard the already ongoing peroxidation because their reaction with peroxyl radicals is too slow to compete with the chain-propagation, thus, neither ERN nor SFN are radical-trapping (chain-breaking) antioxidants [17,18].

According to our best knowledge, there is no information about the antioxidant activity of SFN and ERN at temperatures higher than 40 °C including thermal behaviour of this class of compounds at elevated temperature, i.e., under conditions similar to those in standard cooking in the presence of lipids as food constituents which are the most sensitive to oxidation.

We selected linolenic acid (98%, *all*-*cis*-9,12,15-Octadecatrienoic acid, LNA) as an example of highly unsaturated lipid C18:3, and sunflower oil (SUN) as an example of edible oil frequently used in food preparation. The main fatty acids found in SUN were: palmitic acid (6.2% ± 0.5%), oleic acid (33.4 % ± 2.0%), stearic acid (4.8% ± 0.4%), linoleic acid (55.5% ± 3.4%), and linolenic acid (0.11% ± 0.04%), see details of GC analysis in Supplementary material (Figures S1 and S2 and Table S1). We used Differential Scanning Calorimetry in non-isothermal mode, with small 5-10 mg samples heated with heating rate 2 K/min for SUN and 2.5 K/min for LNA under oxygen flow 6 L/h (experimental details are included in the Supporting Material). Under such conditions the recorded DSC plots represent thermal effects of formation of hydroperoxides (primary products of oxidation) [19], see Figure 2. The DSC curves recorded for oxidation of pure (neat) LNA and SUN indicate that those two lipids have different oxidative stability: oxidation of LNA starts at ca. 90 °C while oxidation of SUN starts at temperature 140 °C (see vertical dotted lines in both panels of Figure 2). In the Supplementary Material the kinetic parameters calculated by iso-conversional method are presented in details: *E*_a_ for oxidation of pure LNA (79 ± 5 kJ/mol) and overall rate constant, *k*, for its oxidation at 50 °C (i.e., during the lag phase) is 7.3 × 10^−3^ min^−1^ and does not change for oxidation of LNA containing ERN or SFN. For sunflower oil the kinetics are quite different: *E*_a_ for oxidation of pure SUN is 103 ± 4 kJ/mol and increases to 119 ± 8 kJ/mol in the presence of 10 mM ERN or 5 mM SFN. Similarly, *k* calculated at 100 °C (lag phase) for oxidation of pure SUN is 5.8 × 10^−3^ min^−1^ but in the presence of 10 mM ERN is almost two-fold smaller (2.9 × 10^−3^ min^−1^), see Supplementary Material (Tables S2–S9 and Figures S3–S9). Complete data will be presented and discussed (together with kinetics of oxidation of other lipid matrices) in a full length article.

In this communication we limit our observations to the slowest β = 2 and 2.5 K/min, when the conditions are relatively close to thermal equilibrium. Non-isothermal experiments allow for a direct comparison of thermal behaviour of two lipid matrices (LNA and SUN) regardless of their different oxidative stability, parametrized as different temperatures of the start of oxidation. The addition of antioxidant or retardant of oxidation should enhance the oxidative stability (increasing lag phase, inhibition phase) and during the non-isothermal oxidation such lag phase should be manifested as the increasing temperature when the oxidation starts, as we observed many times in our previous works with lipids containing phenolic antioxidants like 2,6-di-*tert*-butyl-4-methylphenol (BHT) or tocopherol [20,21,22,23]. However, the thermal behaviour of LNA containing SFN or ERN (at concentrations 1–10 mM) presented in Figure 2a indicates that these two isothiocyanates do not improve the oxidative stability of polyunsaturated lipid because extrapolated start of oxidation is usually at ca. 90 °C (for 10 mM SFN the oxidation starts at even lower temperature). In contrast, sunflower oil that is resistant towards oxidation up to temperature 140 °C is additionally stabilized when SFN or ERN are added, see Figure 2b. For 10 mM concentration of ERN, the prolongation of oxidative stability of SUN is ca. 5 °C and taking into account the non-isothermal character of the experiment, such difference is a clear demonstration of antioxidant contribution of SFN and ERN to the overall kinetics of thermal oxidation of SUN.

The first hypothesis, that ITCs could be activated by the products of SUN decomposition, was excluded because both LNA and SUN produce very similar lipidperoxyl radicals as intermediates and similar hydroperoxides as primary products of autoxidation. We also excluded the explanation that the antioxidant effect is due to the decomposition of the isothiocyanate functional group, because alkyl isothiocyanates are stable and -N=C=S is not eliminated during rather harsh conditions, as evidenced by GC analysis [24], isothiocyanates survive high temperature of GC injector (280 °C) and temperature of GC column (increasing up to 220 °C). Thus, the functionality other than -N=C=S must be responsible for the observed “antioxidant activation” of SFN and ERN at higher temperatures, and we put forward the hypothesis that CH_3_(S=O)- and CH_3_S- groups are responsible for the observed antioxidant action of both ITCs at temperatures close to 140 °C. In our investigations we were inspired by the works on the antioxidant action of diallyl thiosulfinate (allicin—a component of garlic and onion) published recently by Ingold and Pratt [25,26]. Following the excellent review by Block [27], Ingold and Pratt re-vitalized the idea based on older observation of the antioxidant action of sulfoxides in tetralin made by Koelewijn and Berger [28] who explained their inhibitory effect due to formation of transient sulfenic acids during thermal decomposition of sulfoxides via Cope-type elimination (Equation (1)).

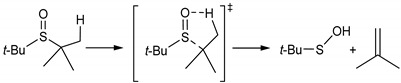
(1)

The rate constants for unimolecular decomposition of di-*tert*-butylsulfoxide is 1.3 × 10^−5^ s^−1^ at 60 °C and for less reactive di-*n*-dodecylsulfoxide is 10^−5^ s^−1^ at 130 °C [28]. Koelewijn and Berger also measured the rate constants for reaction of sulfenic acids with alkylperoxyl radicals (Equation (2)):

R-SOH + R’OO^•^ → non-radical products (2)

to be higher than 10^7^ M^−1^⋅s^−1^ at 60 °C. Sulfenic acids are transient species and if peroxyl radicals are not accessible, they undergo self-condensation to thiosulfinates (here, S-methyl methanethiosulfinate, Equation (3)):

2CH_3_SOH → CH_3_(S=O)SCH_3_ + H_2_O (3)

S-methyl methanethiosulfinate easily decomposes via Cope-type elimination (the process is much faster for thiosulfinates than for dialkylsulfoxides) into another sulfenic acid and thioformaldehyde (Equation (4)) [29,30,31]:

CH_3_(S=O)SCH_3_ → CH_3_SOH + H_2_C=S (4)

therefore, this is an additional chance to trap peroxyl radicals because sulfenic acid is “recovered” as a second generation of intermediates of thermal decomposition of sulfoxides.

Another possible explanation of antioxidant behaviour of SFN at elevated temperature is direct thermolysis, with formation of methylsulfinyl radicals CH_3_SO^•^ that are able to scavenge peroxyl radicals. Methylsulfinyl radicals can be also produced from sulfenic acid reacting with peroxyl radicals (Equation (2) modified to the form: CH_3_SOH + R’OO^•^ → CH_3_SO^•^ + R’OOH). The analogous process was described by Pratt et al. [26], who studied the mechanism of antioxidant action of S-propyl propanethiosulfinate, C_3_H_7_(S=O)SC_3_H_7_, as saturated analogue of allicin and other secondary metabolites of plants belonging to *Allium* *genus*. They computed thermodynamic parameters for model methanesulfenic acids undergoing subsequent reactions with methylsulfinyl radicals as reactive intermediates able to trap alkylperoxyl radicals (Equation (5)).

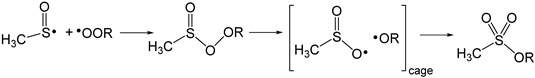
(5)

The above mechanism explains the antioxidant action of allicin analogues at low temperatures, but the same chemistry should be also valid for the antioxidant action of sulforaphane at temperatures above 140 °C.

Similar antioxidant behaviour of both ITCs, regardless of different oxidation state of sulfur atom in ERN and SFN, is not surprising because at high temperature and in the presence of O_2_, sulfides can be easily oxidised to sulfoxides. Moreover, formation of unstable sulfenic acid was also observed for cysteine in the systems with induced stress (elevated temperature is not necessary) [32]. The electron withdrawing group at β position (β-EWG) greatly facilitates the process, but Cope-type elimination not assisted by β-EWG was also observed by Kubec et al. [33] for S-methylcysteine sulfoxide heated at 120 °C for 1 h (complete conversion in the presence of 10% of water, for the dry compound the process was “somewhat slower”). On the other side, generation of methylsulfinyl radical was proposed by Jin et al. [24] on the basis of careful analysis of the products of thermal decomposition of sulforaphane in water at 50–100 °C. Both mentioned works considered decomposition in a polar environment, thus, we decided to check the products of thermal decomposition of neat ERN and SFN at temperatures of 100 and 160 °C.

The results of the GC/MS analysis are presented in Table 1 and Figure 3. Indeed, for both, ITCs we found three compounds that are the same as described by Jin et al. [24] for thermal decomposition of SFN, that is a proof that ERN readily undergoes oxidation to SFN.

All three compounds, **3A**, **3B** and **3C**, were found in the samples containing SFN and ERN heated at 160 °C, but S-methyl methylthiosulfonate (**3C**) was not detected at lower temperatures, perhaps because of a slower reaction at 100 °C, where the amount of **3C** was below the detection limit. Moreover, SFN undergoes further processes: dehydratation and formation of **3A** (we detected both *E*/*Z* isomers), product of β-elimination **3B** (second product of this reaction, sulfenic acid, was not detected because of its instability) and cleavage of S-C bond with generation of methylsulfinyl radical which, after recombination with methylsulfanyl radical (CH_3_S^•^), gives S-methyl methanethiosulfinate, further oxidized to **3C**. Compound **3A** was reported by Papi et al. [18] as exhibiting selective cytotoxic/apoptotic activity, however, the same authors noticed that regardless of the reaction with diphenylpicrylhydrazyl radicals, **3A** was not a good chain-breaking inhibitor of low temperature autoxidation of styrene and methyl linoleate.

In conclusion, our experiments with thermal oxidation of two model lipids indicate that erucin and sulforaphane are kinetically neutral below 100 °C but at elevated temperatures these isotiocyanates decompose (with kinetically significant rates) to sulfenic acids and/or methylsulfinyl radicals that are good radical trapping agents, resulting in better oxidative stability of the lipid system. Thus, ITCs are examples of biocompounds which (in contrast to phenolic antioxidants) become chain-breaking antioxidants above 120 °C, that is, at temperatures corresponding to the frying process. A similar effect of inhibition of autoxidation at temperatures above 140 °C by SFN and ERN was observed by us during thermal oxidation of soy lecithin. Detailed description with the kinetic parameters for spontaneous and inhibited oxidation and comparison for LNA, SUN and soy lecithin will be described in a full length article.

## Figures and Tables

**Figure 1:**
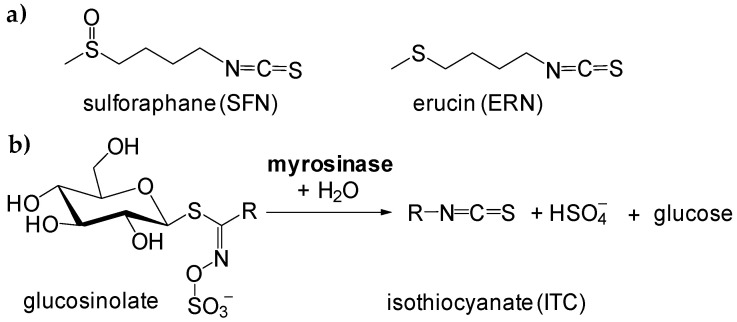
Structures of sulforaphane and erucin (**a**), and reaction scheme of formation of free isothiocyanates (ITCs) during hydrolysis of glucosinolates in the presence of myrosinase (**b**).

**Figure 2:**
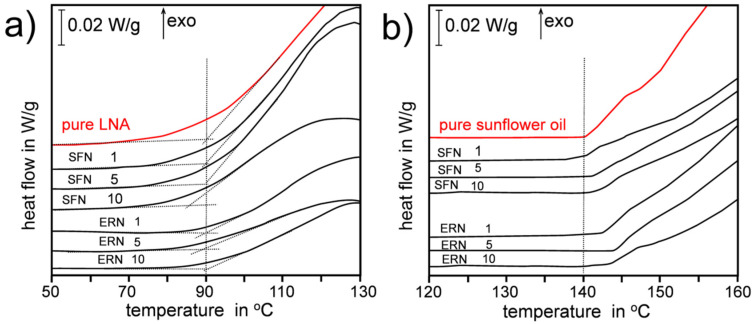
Thermal effects of non-isothermal oxidation (heating rate β = 2.5 K/min) of pure linolenic acid (LNA) and LNA containing 1, 5 and 10 mM sulforaphane (SFN)/erucin (ERN) (panel **a**). Thermal effects of non-isothermal oxidation (β = 2.0 K/min) of pure SUN and SUN containing 1, 5 and 10 mM SFN/ERN (panel **b**). The DSC curves were vertically shifted for clarity. The number above each DSC curve denotes SFN/ERN concentration (in mM). Vertical dotted lines at 90 °C and 140 °C are introduced as a guide to the eye to observe a shift of temperatures of start of oxidation.

**Figure 3:**
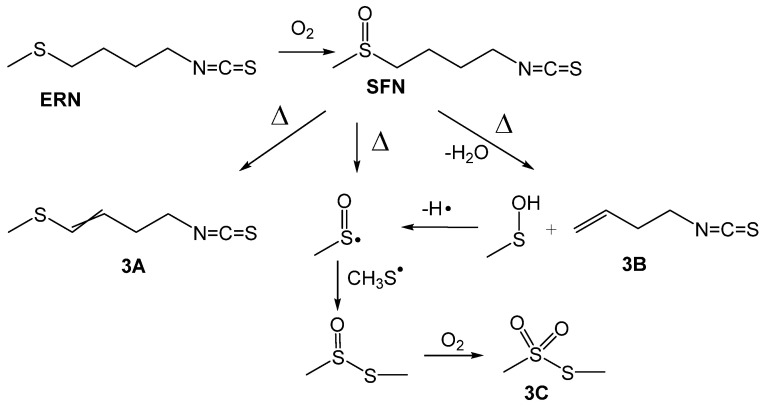
Proposed paths of thermal oxidative conversion of erucin (ERN) to sulforaphane (SFN) and subsequent decomposition to the products detected by GC/MS and showed in Table 1.

**Table 1:** Names, retention times and mass spectral data for three products of thermal decomposition of neat SFN/ERN at temperatures 100 °C and 160 °C.

Compound, Symbol, R_T_^1^	Experiment ^5^	MS Spectral Data *m*/*z* (Relative Intensity)
4-methylthio-3-butenyl isothiocyanate ^2^ **3A**, R_T_ = 34.2 min	SFN(100 °C, 160 °C), ERN(160 °C)	161 [(M+2)^+^, 9], 87 (22), 85 (24), 72 (42), 61 (100), 45 (34)
but-3-enyl isothiocyanate ^3^ **3B**, R_T_ = 11.6 min	SFN(100 °C, 160 °C), ERN(160 °C)	113 (M^+^, 50), 85 (10), 72 (100), 55 (30), 39 (71)
S-methyl methylthiosulfonate ^4^ **3C**, R_T_ = 10.5 min	SFN(160 °C), ERN(160 °C)	128 [(M+2)^+^, 8], 126 (M^+^, 73), 111 (11), 81 (5), 79 (50), 64 (40), 47 (10), 45 (20)

^1^ R_T_ = retention time. ^2^ Identified basing on NIST Mass Spectrometry Data Center, SRD1A, NIST MS numbers 5649 and 414629 (accessed on 14.08.2020). ^3^ Identified basing on NIST Mass Spectrometry Data Center, SRD 69, NIST MS number 414530 (accessed on 14.08.2020). ^4^ Identified basing on NIST Mass Spectrometry Data Center, SRD 69, NIST MS number 236079 (accessed on 14.08.2020). ^5^ This column indicates in which experiment the compounds were detected for thermal decomposition of SFN/ERN.

## Supplementary Materials

The following are available online at https://www.mdpi.com/2076-3921/9/11/1090/s1, Scheme S1: pathway of the synthesis of ERN and SFN; Figure S1: GC chromatogram of FAME Mix RM-3 reference mixture; Figure S2: GC chromatogram of FAME obtained from the sunflower oil; Table S1: Relative FAME composition obtained from the sunflower oil; Tables S2–S9 and Figures S3–S9: Experimental procedures (conversion of SUN into fatty acid methyl esters, analysis of fatty acids profile, synthesis of sulforaphane and erucin, NMR and MS analysis of ERN and SFN, experimental procedure of thermal degradation of ERN/SFN and GC-MS analysis of the products, description of DSC measurements, methodology of calculation of kinetic parameters of oxidation) the values of activation energy (*E*_a_), pre-exponential factor *Z*, and overall rate constant (*k*) calculated for oxidations of pure LNA/SUN and for oxidations of LNA/SUN containing ERN/SFN; references.
