# Supplementary material for: A Lesson Learnt from Food Chemistry—Elevated Temperature Triggers the Antioxidant Action of Two Edible Isothiocyanates: Erucin and Sulforaphane
Source: Antioxidants (Basel). 2020 Nov 6;9(11):1090. doi: 10.3390/antiox9111090 (PMC7694611; doi:10.3390/antiox9111090)
Supplement: Supplementary file 1 [file antioxidants-09-01090-s001.pdf]

Supplementary Material

A lesson learnt from food chemistry - elevated temperature triggers the antioxidant action of two edible isothiocyanates: erucin and sulforaphane

Jakub Cedrowski,[a] Kajetan Dabrowa,[b] Agnieszka Krogul-Sobczak,[a] Grzegorz Litwinienko[a*]

[a] University of Warsaw, Faculty of Chemistry, Pasteura 1, 02-093 Warsaw, Poland.

[b] Polish Academy of Sciences, Institute of Organic Chemistry, Kasprzaka 44/52, 01-224 Warsaw, Poland.

*Corresponding author (e-mail: litwin@chem.uw.edu.pl)

TABLE OF CONTENTS

Title	Page
**EXPERIMENTAL PROCEDURES**	**S-3**
**Scheme S1.** Pathway of the synthesis of ERN and SFN.	**S-3**
**Figure S1.** GC chromatogram of FAME Mix RM-3 reference mixture.	**S-4**
**Figure S2.** GC chromatogram of FAME obtained from the sunflower oil.	**S-4**
**Table S1.** Relative FAME composition obtained from the sunflower oil.	**S-4**
**METHODOLOGY OF CALCULATION OF KINETIC PARAMETERS OF OXIDATION**	**S-5**
**Table S2.** Summary of the kinetic parameters (E_a, Z, and k at 100, 140 and 150°C) calculated for oxidation. Results were collected from Tables S3-S9.	**S-5**
**Figure S3.** Plot of logβ versus $1000T_e^{-1}$ (equation S1) for oxidation of pure SUN.	**S-6**
**Table S3.** Temperatures of extrapolated start of oxidation (T_e) statistical parameters of equation S1 (logβ=$a/T_e + b$), and kinetic parameters for oxidation of pure SUN.	**S-6**
**Figure S4.** Plot of logβ versus $1000T_e^{-1}$ (equation S1) for oxidation of SUN + 1.0 mM SFN.	**S-7**
**Table S4.** Temperatures of extrapolated start of oxidation (T_e) statistical parameters of equation S1 (logβ=$a/T_e + b$), and kinetic parameters for oxidation of SUN + 1.0 mM SFN.	**S-7**
**Figure S5.** Plot of logβ versus $1000T_e^{-1}$ (equation S1) for oxidation of SUN + 5.0 mM SFN.	**S-8**
**Table S5.** Temperatures of extrapolated start of oxidation (T_e) statistical parameters of equation S1 (logβ=$a/T_e + b$), and kinetic parameters for oxidation of SUN + 5.0 mM SFN.	**S-8**
**Figure S6.** Plot of logβ versus $1000T_e^{-1}$ (equation S1) for oxidation of SUN + 10.0 mM SFN.	**S-9**
**Table S6.** Temperatures of extrapolated start of oxidation (T_e) statistical parameters of equation S1 (logβ=$a/T_e + b$), and kinetic parameters for oxidation of SUN + 10.0 mM SFN.	**S-9**
**Figure S7.** Plot of logβ versus $1000T_e^{-1}$ (equation S1) for oxidation of SUN + 1.0 mM ERN.	**S-10**
**Table S7.** Temperatures of extrapolated start of oxidation (T_e) statistical parameters of equation S1 (logβ=$a/T_e + b$), and kinetic parameters for oxidation of SUN + 1.0 mM ERN.	**S-10**
**Figure S8.** Plot of logβ versus $1000T_e^{-1}$ (equation S1) for oxidation of SUN + 5.0 mM ERN.	**S-11**
**Table S8.** Temperatures of extrapolated start of oxidation (T_e) statistical parameters of equation S1 (logβ=$a/T_e + b$), and kinetic parameters for oxidation of SUN + 5.0 mM ERN.	**S-11**
**Figure S9.** Plot of logβ versus $1000T_e^{-1}$ (equation S1) for oxidation of SUN + 10.0 mM ERN.	**S-12**
**Table S9.** Temperatures of extrapolated start of oxidation (T_e) statistical parameters of equation S1 (logβ=$a/T_e + b$), and kinetic parameters for oxidation of SUN + 10.0 mM ERN.	**S-12**
**REFERENCES**	**S-13**

EXPERIMENTAL PROCEDURES

Synthesis of sulforaphane and erucin

The synthetic procedures will be described in full length article. Erucin and D,L-sulforaphane were prepared by synthetic route described in Scheme **S1**.

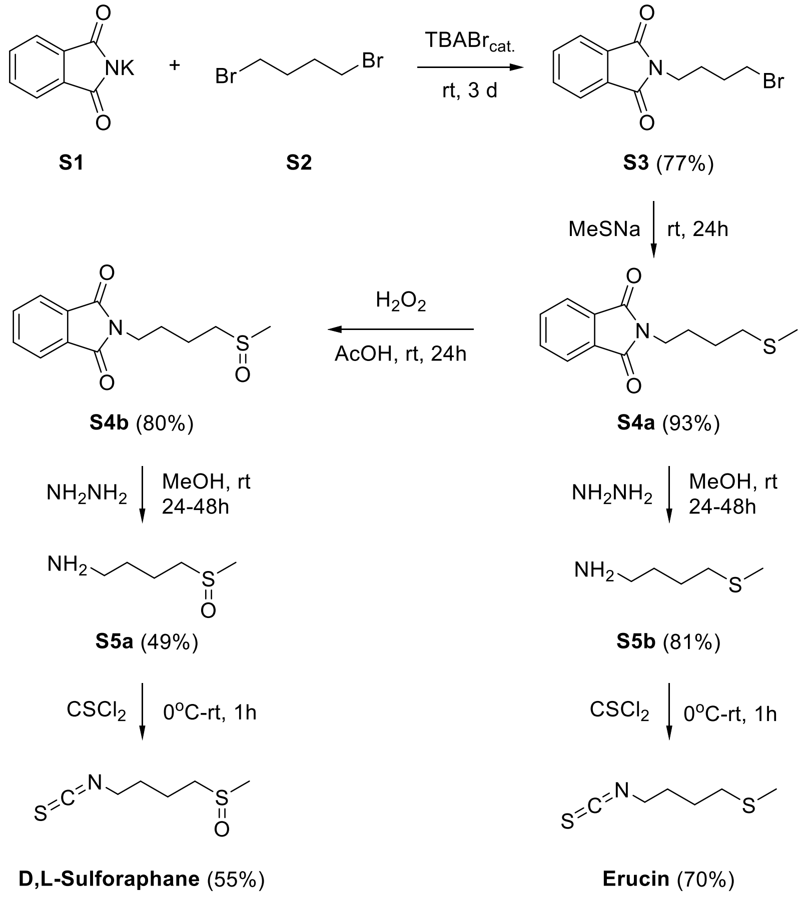

Scheme S1. Pathway of the synthesis of ERN and SFN. Full description of the procedures together with NMR spectra for each step and MS spectra for SFN and ERN will be presented in full length article.

Conversion of the sunflower oil into fatty acid methyl esters. Sunflower oil (edible oil) was converted into fatty acid methyl esters (FAME) by the procedure developed by Matcalfe et al. [1] using boron trifluoride as catalyst. Methanolic sodium hydroxide (5 ml of 0.5 M) was added to 1 mL of oil and heated in round bottom flask with magnetic stirrer for 20 min under nitrogen. Then, 5 mL of 15% boron trifluoride in methanol was added in order to methylate the samples and the mixture was heated and stirred for additional 5 min. After cooling the flask, hexane (5 ml) and brine were added, the mixture was shaken and hexane fraction was separated, and dried over Na_2SO_4. After evaporation the sample was dissolved in toluene and analysed by GC. We used FAME Mix RM-3 as the reference mixture of fatty acid methyl esters.

Analysis of fatty acids profile. Gas chromatography was performed using GCMS-QP2010 Ultra gas chromatograph (GC) coupled to QP-5000 (Shimadzu) single quadrupole mass spectrometer (MS); GC/MS was equipped with AOC-5000 autosampler (Shimadzu). Separation of the studied compounds was carried out with HP-5ms (Agilent) chemically bonded fused silica capillary column: (30m x 0.25mm, 0.25 µm film thickness). Carrier gas (He) was delivered at a flow rate of 9.5 ml/min, split ratio was 10 and the pressure was maintained constantly. Chromatographic conditions were as follows: injector temperature 270°C; temperatures of the MS transfer line and ion source were set to 270°C. Temperature was programmed as follows: initially 50°C maintained for 1 min, then raising with the rate 20°C/min to 150°C, maintained for 5 min, then raising with the rate 1°C/min to 260 °C ,

maintained for 2 min; analysis was completed in 123 minutes. MS was equipped with electron impact (EI) ion source (70 eV) and the spectrum was acquired in the total ion current (TIC) mode in the mass range 35 - 450 m/z, solvent cut was 5 min. GCMSsolution 2.53 (Shimadzu) software was used for data analysis.

NMR analysis of erucin and sulforaphane. ^{1}H NMR and ^{13}C NMR spectra were recorded on Bruker Mercury 500 instrument at 500 and 125 MHz. NMR signals were assigned with the help of DEPT, COSY, HMBC, HMQC, and NOESY experiments. Proton and carbon chemical shifts are reported in ppm (δ) ($CDCl_3$, ^{1}H NMR δ = 7.26 and ^{13}C NMR δ = 77.26). *J* coupling constants values are reported in Hz.

MS measurements. High resolution mass spectra (HRMS) were recorded using ESI-TOF (SFN) or EI (ERN) techniques.

**Erucin (ERN)**	**^{1}H NMR** (500 MHz, $CDCl_3$): δ 3.55 (t, *J* = 6.4 Hz, 2H), 2.53 (t, *J* = 7.0 Hz, 2H), 2.10 (s, 3H), 1.85–1.78 (m, 2H), 1.77–1.70 (m, 2H).<br>**^{13}C NMR** (125 MHz, $CDCl_3$): δ 130.4, 44.9, 33.4, 29.0, 26.0, 15.6.<br>**HRMS EI** (m/z): Calc. for $C_6H_{11}NS_2$: 161.0333 $[M]^+$, found: 161.0331.
**D,L-Sulforaphane (SFN)**	**^{1}H NMR** (500 MHz, $CDCl_3$): δ 3.59 (td, *J* = 6.4, 1.0 Hz, 2H), 2.71 (tt, *J* = 13.1, 6.9 Hz, 2H), 2.58 (s, 3H), 1.98–1.82 (m, 4H).<br>**^{13}C NMR** (125 MHz, $CDCl_3$): δ 131.2, 53.6, 44.8, 38.9, 29.1, 20.2.<br>**HRMS ESI** (m/z): Calc. for $C_6H_{11}OS_2Na$: 200.0180 $[M+Na]^+$, found: 200.0185.

DSC measurements. The calorimetric measurements were carried out using a DSC apparatus Du Pont 910 differential scanning calorimeter with Du Pont 9900 thermal analyzer and a normal pressure cell. The apparatus was calibrated with high purity indium. The oxidations were performed under oxygen flow 6 dm^3/min, 3-5 mg samples were heated from 50 to 200°C in open pan with linear heating rate 2 or 2.5 K/min, and as a reference material an empty aluminum pan was used. Calorimetric signals were recorded with TA Instruments software (General V4.01).

Preparation of samples for DSC analysis. Portion of LNA or SUN (0.8 – 1 g) was dissolved in 10 ml of methanol (LNA) or methylene chloride (SUN), and the appropriate volume of ITC (isothiocyanate: ERN or SFN) solution in methanol/methylene chloride was added, then the solvent was removed with rotary evaporator at 40°C and under 50 hPa until a constant mass of the sample was obtained, giving the final concentrations of ITCs 1.0 – 10.0 mM.

Thermal decomposition of ITCs and GC-MS measurements. Samples of ITC (1-2 mg) were placed (under air) in ~ 2ml glass ampoule along with a tiny drop of water and sealed. The ampoule with the sample was heated at 100°C and 160°C for 5h and 30min respectively. The reaction mixture was then cooled down in -70°C (dry ice/acetone bath), the head of ampoule was broken and the content was dissolved in 500 µl of methylene chloride (DCM). The solution was moved to vial containing 1 ml of water. The DCM layer phase was subjected to GC-MS analysis directly. GC-MS analysis was performed with HP5 capillary column (0.32 mm diameter, 30 m length, 0.25 µm thickness, Hewlett-Packard) with GC-17A Ver.3 Shimadzu and GCMS-QP5050A Shimadzu mass detector. Mass spectrum was obtained by electron impact (EI) ionization mode, scanning from 33 m/z to 350 m/z. The flow rate of carrier gas (He) was 1.5 ml/min, and the split ratio was 5:1. Inlet temperature was 210°C and oven temperature program was as follows: an initial step starts at 40°C (isothermal for 5 min); raising with the rate 3°C/min to 220°C.

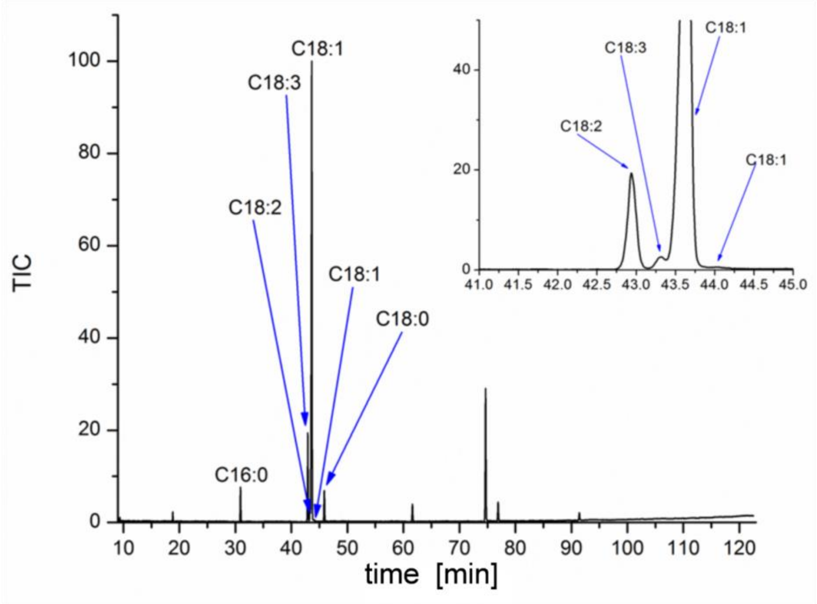

Figure S1. GC chromatogram of FAME Mix RM-3 reference mixture.

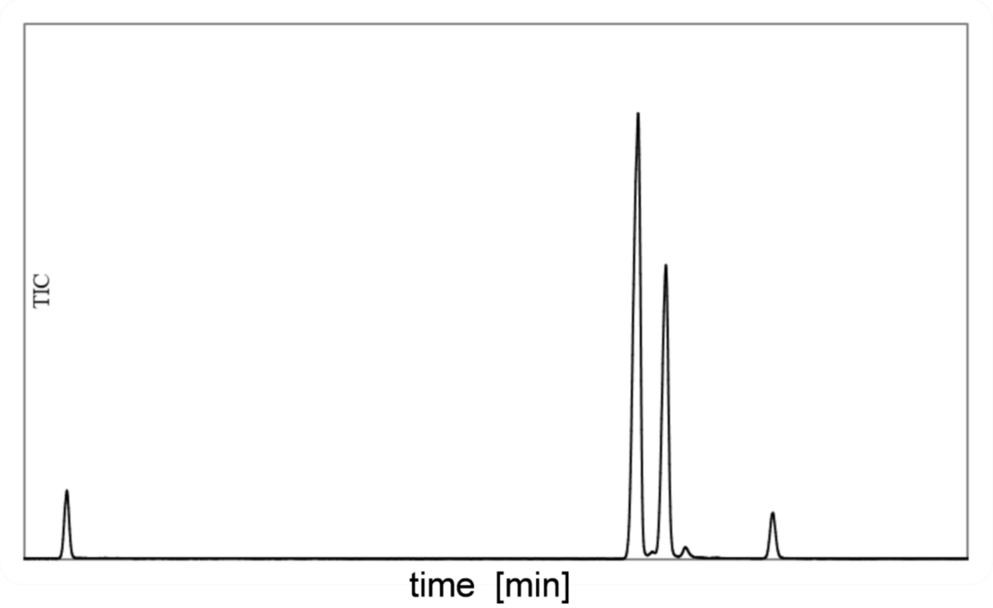

Figure S2. GC chromatogram of FAME obtained from the sunflower oil. Conditions of analysis are the same as described in Figure S1.

Table S1. Relative FAME composition obtained from the sunflower oil. The symbol for an acid includes the total number of carbons and double bonds (e.g., 18:2).

**Sample**	**C16:0**	**C18:0**	**C18:1[2]**	**C18:1[2]**	**C18:2**	**C18:3**
Retention time (min)	30.9	45.8	43.5	44.0	43.0	43.3
Reference	4.70%	4.79%	76.65%	0.050%	12.88%	0.92%
RSD[1]	0.22%	0.14%	2.90%	0.009%	0.25%	0.25%
Sunflower	6.18%	4.76%	32.55%	0.86%	55.53%	0.11%
RSD	0.45%	0.38%	1.99%	0.17%	3.35%	0.04%

[1] Standard deviation calculated for 3 injections. [2] E/Z isomers.

Methodology of calculation of kinetic parameters of oxidation

The values of activation energy (E_a) and frequency factor Z for oxidation processes were calculated by the Ozawa-Flynn-Wall's method [2-4]. This method has been successfully applied by us [5-7] and others[8-10] for studies of the kinetics of non-isothermal oxidation of hydrocarbons and lipids. In our previous publications [11-13] we interpreted the shape of DSC curves of thermal effect of lipids and hydrocarbons oxidation and we proved that T_e parameter corresponds to constant degree of conversion (start of oxidation), therefore, thermoanalytical procedures based on iso-conversional methods (like Ozawa-Flynn-Wall method) can be applied for calculations of the kinetic parameters of oxidation.

Increase of heating rate (β) causes a shift of temperature of start of oxidation (T_e) to higher values. Thus, after several experiments (each with fresh sample) for different β, the linear dependence is obtained:

$$\log\beta = a \times \mathrm{T_e}^{-1} + b \qquad \text{(S1)}$$

with the slope $a = -0.456E_a/R$ and intersection $b=-2.315 + log(ZE_a/R)$, where R is gas constant (8.314 [$Jmol^{-1}K^{-1}$]). E_a calculated from the slope a and this overall (global) parameter is a sum of three activation components of this chain reaction: propagation E_p, initiation E_i, termination E_t:

$$E_a = E_p + 0{,}5E_i - 0{,}5E_t \qquad \text{(S2)}$$

Determination of the kinetic parameters for non-isothermal autoxidation should be based on the start of oxidation (T_e) as thermal effect at this point of DSC curve is not contaminated by heat of decomposition of hydroperoxides formed during autoxidation. Addition of chain-breaking antioxidant inhibits the propagation step and has an impact on the rate of formation of primary products. Thus, monitoring the changes of T_e temperature allow to calculate the overall kinetic parameters of inhibited and uninhibited oxidation [12,14,15].

The rate constants, k, for overall oxidation process were calculated for temperatures 100°C, 140°C, and 150°C from the Arrhenius equation:

$$k = Z\, exp(-E_a/RT) \qquad \text{(S3)}$$

For every series (pure SUN, SUN containing 1, 5, 10 mM SFN, and SUN containing 1,5,10 mM ERN) logβ was calculated, then plot of logβ versus 1000/T_e dependence was drawn (see Figures S3-S9), afterwards, using linear equation (Equation S1), factors a and b were obtained (equations S2, S3).

Table S2. The values of the apparent activation energy (E_a), pre-exponential factor Z, and overall rate constant (k) at 100, 140 and 150°C calculated for oxidation of sunflower oil (SUN) containing two isothiocyanates (ITCs) sulforaphane and erucin at concentrations 1.0-10.0 mM. **The results presented in this table are collected from Tables S3-S9**.

C_{ITC} [mM]	E_a [kJ/mol]	Z [min^{-1}]	k (100°C) [min^{-1}]	k (140°C) [min^{-1}]	k (150°C) [min^{-1}]
pure SUN	104 ± 4	1.75×10^{12}	5.87×10^{-3}	0.148	0.301
sulforaphane					
1.0	115 ± 5	4.53×10^{13}	3.76×10^{-3}	0.135	0.295
5.0	119 ± 8	1.51×10^{14}	3.48×10^{-3}	0.142	0.322
10.0	114 ± 11	3.55×10^{13}	4.83×10^{-3}	0.166	0.362
erucin					
1.0	115 ± 4	4.01×10^{13}	3.58×10^{-3}	0.128	0.282
5.0	114 ± 9	3.75×10^{13}	3.72×10^{-3}	0.132	0.289
10.0	119 ± 7	1.57×10^{14}	2.94×10^{-3}	0.124	0.281

For oxidation of linolenic acid E_a =79±5kJ/mol, $Z = 4.5 \times 10^{10}$ min^{-1} and calculated $k_{50°C}$ was 7.3×10^{-3} min^{-1}. Presence of SFN or ERN did not change the rates of oxidation (values $k_{50°C}$ were scattered within the range from 0.0052 to 0.012 min^{-1}) and $k_{100°C}$ (ie., above T_e ~90°C) indicated apparent prooxidative effects of SFN and ERN, those effects will be presented and discussed in full length article).

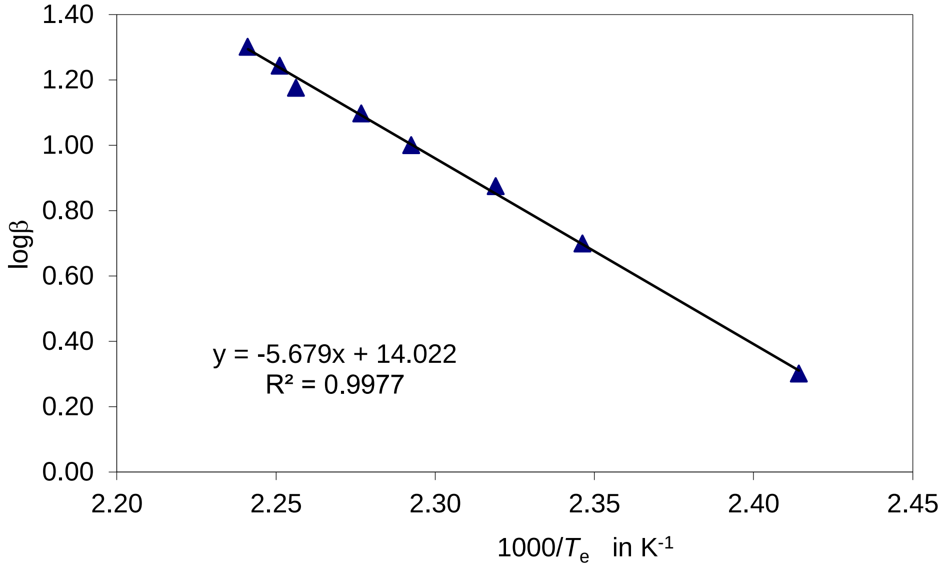

Figure S3. Plot of logβ versus $1000T_e^{-1}$ (equation S1) for oxidation of pure sunflower oil.

Table S3. Temperatures of extrapolated start of oxidation (T_e) determined for various heating rates β for oxidation of pure sunflower oil, the parameters of equation S1 ($\log\beta = a/T_e + b$), standard error σ and error for confidence level 90% ($\sigma_{90\%}$), overall activation energy (E_a) and pre-exponential factor (Z), and rate constants (k) calculated from the Arrhenius equation S3 for 100, 140, 150°C.

β [°C/min]	T_e [°C]	Statistical and kinetic parameters			
2.0	141				
5.0	153	a=	-5.6790		
7.5	158	b=	14.0217	k (100°C) [min^{-1}] =	5.87×10^{-3}
10.0	163	R^2=	0.9977	k (140°C) [min^{-1}] =	0.148
12.5	166	σ=	0.112	k (150°C) [min^{-1}] =	0.301
15.0	170	$\sigma_{90\%}$=	0.208		
17.5	171	**E_a** [kJ/mol] =	104±4		
20.0		***Z*** [min^{-1}] =	1.75×10^{12}		

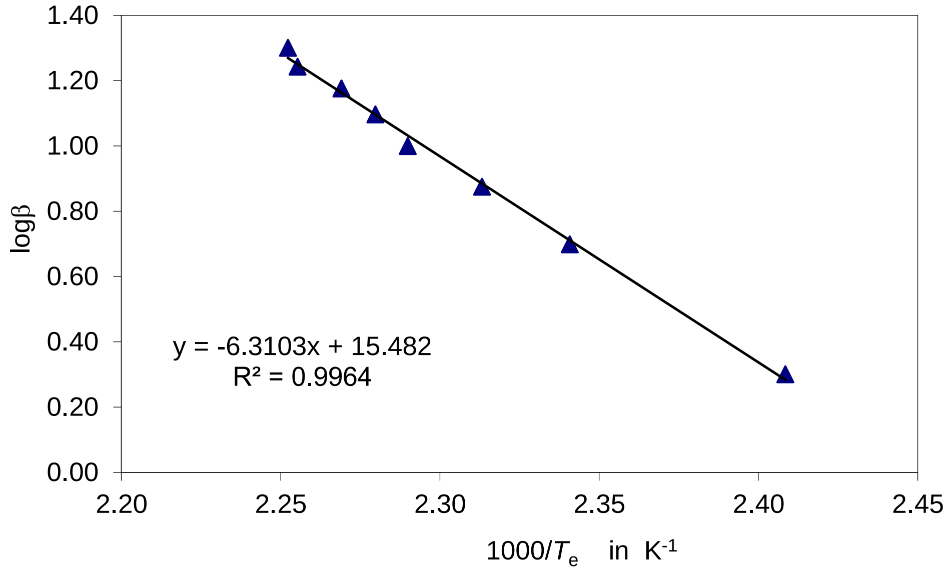

Figure S4. Plot of logβ versus 1000T_e^{-1} (equation S1) for oxidation of sunflower oil containing 1 mM SFN.

Table S4. Temperatures of extrapolated start of oxidation (T_e) determined for various heating rates β for oxidation of pure sunflower oil containing 1 mM SFN, the parameters of equation S1 (logβ=$a/T_e + b$), standard error σ and error for confidence level 90% ($\sigma_{90\%}$), overall activation energy (E_a) and pre-exponential factor (Z), and rate constants (k) calculated from the Arrhenius equation S3 for 100, 140, 150°C.

β [°C/min]	T_e [°C]	Statistical and kinetic parameters			
2.0	142.0				
5.0	154.0	a=	-6.3103		
7.5	159.1	b=	15.4821	k (100°C) [min^{-1}] =	3.76× 10^{-3}
10.0	163.5	R^2=	0.9964	k (140°C) [min^{-1}] =	0.135
12.5	165.5	σ=	0.154	k (150°C) [min^{-1}] =	0.295
15.0	167.5	$\sigma_{90\%}$=	0.287		
17.5	170.2	**E_a** [kJ/mol] =	115± 5		
20.0	170.8	***Z*** [min^{-1}] =	4.53 × 10^{13}		

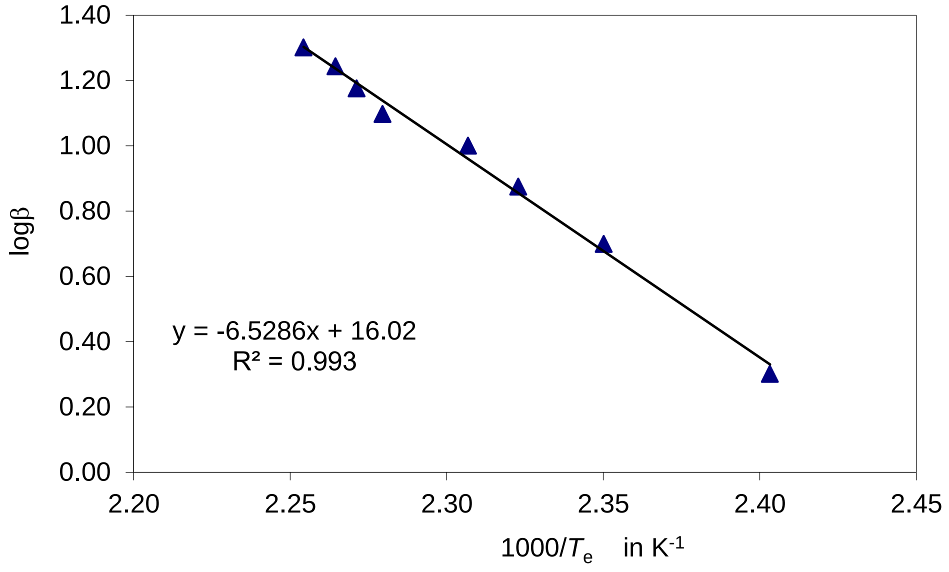

Figure S5. Plot of logβ versus $1000T_e^{-1}$ (equation S1) for oxidation of sunflower oil containing 5 mM SFN.

Table S5. Temperatures of extrapolated start of oxidation (T_e) determined for various heating rates *β* for oxidation of pure sunflower oil containing 5 mM SFN, the parameters of equation S1 ($\log\beta = a/T_e + b$), standard error σ and error for confidence level 90% ($\sigma_{90\%}$), overall activation energy (E_a) and pre-exponential factor (Z), and rate constants (k) calculated from the Arrhenius equation S3 for 100, 140, 150°C.

β [°C/min]	T_e [°C]	Statistical and kinetic parameters			
2.0	142.9				
5.0	152.3	a=	-6.5286		
7.5	157.3	b=	16.0202	k (100°C) [min^{-1}] =	3.48×10^{-3}
10.0	160.3	R^2=	0.9930	k (140°C) [min^{-1}] =	0.142
12.5	165.5	σ=	0.223	k (150°C) [min^{-1}] =	0.322
15.0	167.1	$\sigma_{90\%}$=	0.416		
17.5	168.4	**E_a** [kJ/mol] =	119± 8		
20.0	170.4	***Z*** [min^{-1}] =	1.51×10^{14}		

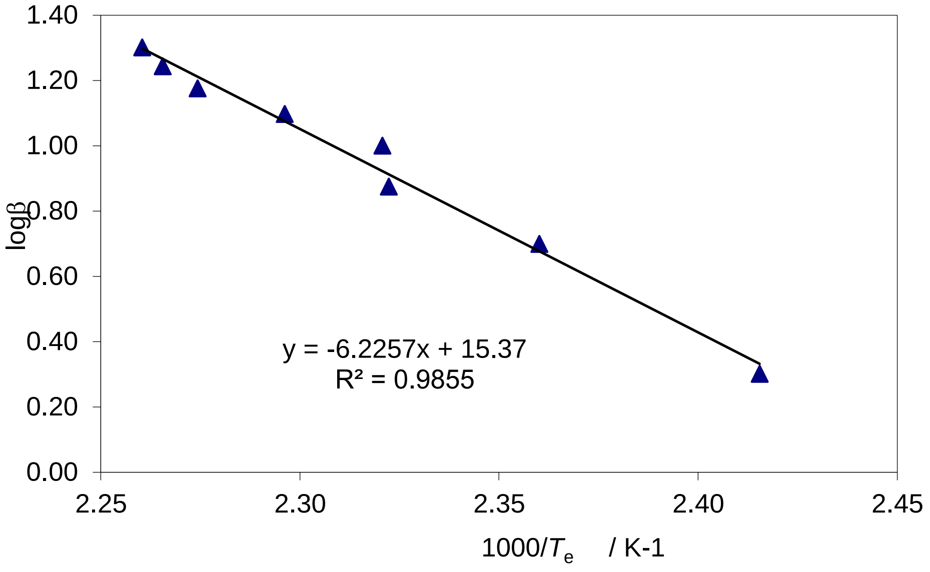

Figure S6. Plot of logβ versus $1000T_e^{-1}$ (equation S1) for oxidation of sunflower oil containing 10 mM SFN.

Table S6. Temperatures of extrapolated start of oxidation (T_e) determined for various heating rates β for oxidation of pure sunflower oil containing SFN, the parameters of equation S1 (log$\beta = a/T_e + b$), standard error σ and error for confidence level 90% ($\sigma_{90\%}$), overall activation energy (E_a) and pre-exponential factor (Z), and rate constants (k) calculated from the Arrhenius equation S3 for 100, 140, 150°C.

β [°C/min]	T_e [°C]	Statistical and kinetic parameters			
2.0	140.8				
5.0	150.5	a=	-6.2257		
7.5	157.4	b=	15.3701	k (100°C) [min^{-1}] =	4.83×10^{-3}
10.0	157.7	R^2=	0.9855	k (140°C) [min^{-1}] =	0.166
12.5	162.3	σ=	0.308	k (150°C) [min^{-1}] =	0.362
15.0	166.5	$\sigma_{90\%}$=	0.572		
17.5	168.2	$\mathbf{E_a}$ [kJ/mol] =	114± 11		
20.0	169.2	$\boldsymbol{Z}$ [min^{-1}] =	3.55×10^{13}		

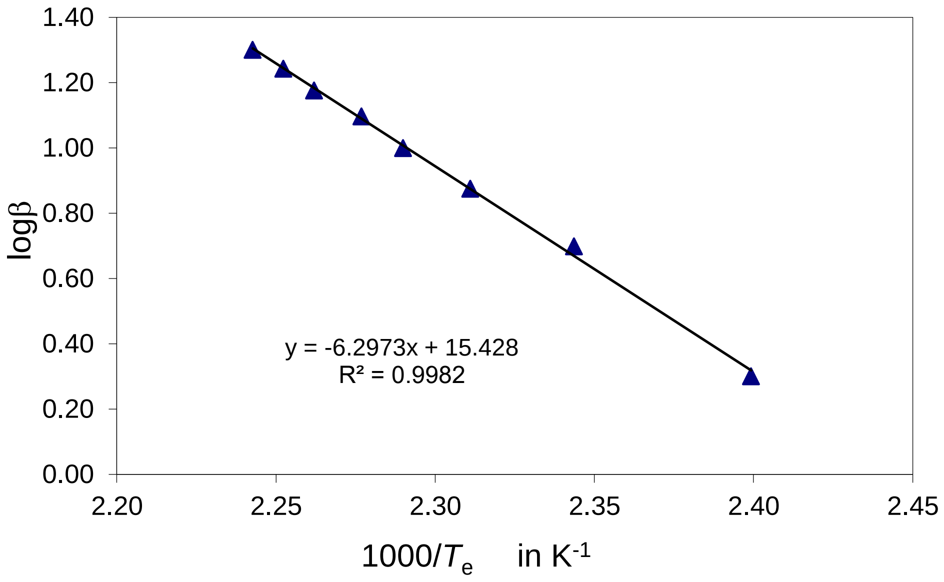

Figure S7. Plot of logβ versus $1000T_e^{-1}$ (equation S1) for oxidation of sunflower oil containing 1 mM ERN.

Table S7. Temperatures of extrapolated start of oxidation (T_e) determined for various heating rates β for oxidation of pure sunflower oil containing ERN, the parameters of equation S1 (logβ=$a/T_e + b$), standard error σ and error for confidence level 90% ($\sigma_{90\%}$), overall activation energy (E_a) and pre-exponential factor (Z), and rate constants (k) calculated from the Arrhenius equation S3 for 100, 140, 150°C.

β [°C/min]	T_e [°C]	Statistical and kinetic parameters			
2.0	143.6				
5.0	153.5	a=	-6.2973		
7.5	159.5	b=	15.4275	k (100°C) [min^{-1}] =	3.58×10^{-3}
10.0	163.5	R^2=	0.9982	k (140°C) [min^{-1}] =	0.128
12.5	166.0	σ=	0.109	k (150°C) [min^{-1}] =	0.282
15.0	168.9	$\sigma_{90\%}$=	0.202		
17.5	170.8	$\mathbf{E_a}$ [kJ/mol] =	115± 4		
20.0	172.7	$\boldsymbol{Z}$ [min^{-1}] =	4.01×10^{13}		

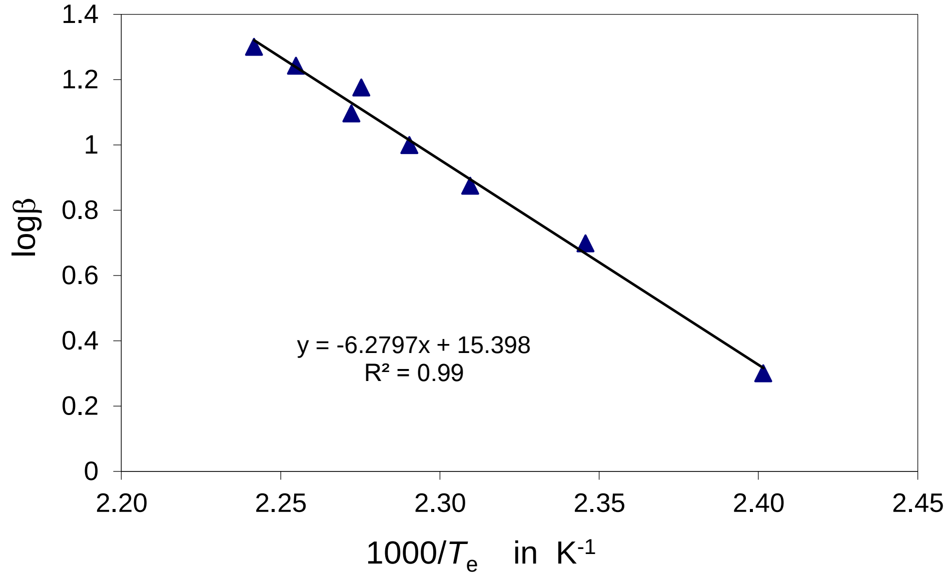

Figure S8. Plot of logβ versus 1000T_e^{-1} (equation S1) for oxidation of sunflower oil containing 5 mM ERN.

Table S8. Temperatures of extrapolated start of oxidation (T_e) determined for various heating rates β for oxidation of pure sunflower oil containing ERN, the parameters of equation S1 (logβ=$a/T_e + b$), standard error σ and error for confidence level 90% ($\sigma_{90\%}$), overall activation energy (E_a) and pre-exponential factor (Z), and rate constants (k) calculated from the Arrhenius equation S3 for 100, 140, 150°C.

β [°C/min]	T_e [°C]	Statistical and kinetic parameters			
2.0	143.2				
5.0	153.1	a=	-6.2797		
7.5	159.8	b=	15.3979	k (100°C) [min^{-1}] =	3.72 × 10^{-3}
10.0	163.4	R^2=	0.9900	k (140°C) [min^{-1}] =	0.132
12.5	166.9	σ=	0.258	k (150°C) [min^{-1}] =	0.289
15.0	166.3	$\sigma_{90\%}$=	0.480		
17.5	170.3	**E_a** [kJ/mol] =	114 ± 9		
20.0	172.9	***Z*** [min^{-1}] =	3.75× 10^{13}		

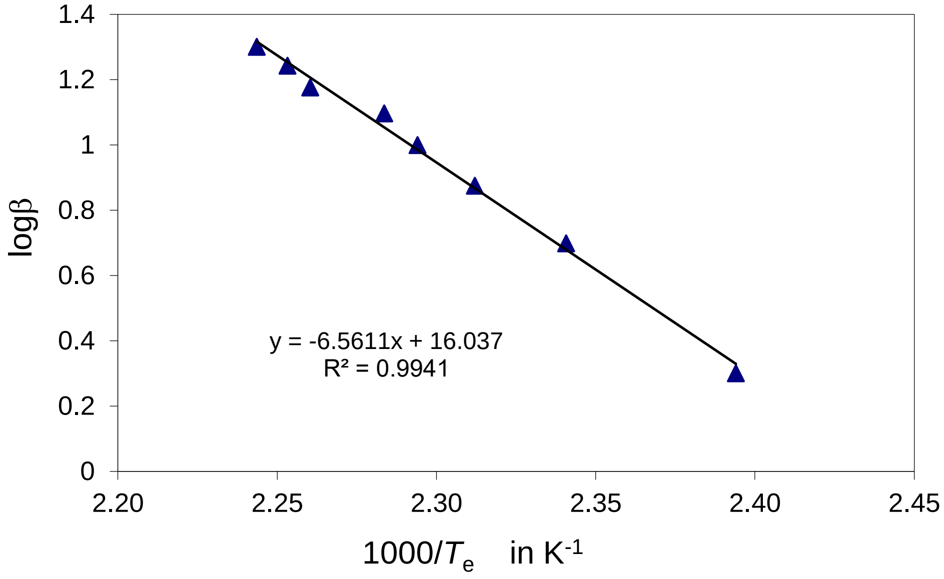

Figure S9. Plot of logβ versus $1000T_e^{-1}$ (equation S1) for oxidation of sunflower oil containing 10 mM ERN.

Table S9. Temperatures of extrapolated start of oxidation (T_e) determined for various heating rates β for oxidation of pure sunflower oil containing ERN, the parameters of equation S1 (logβ=$a/T_e + b$), standard error σ and error for confidence level 90% ($\sigma_{90\%}$), overall activation energy (E_a) and pre-exponential factor (Z), and rate constants (k) calculated from the Arrhenius equation S3 for 100, 140, 150°C.

β [°C/min]	T_e [°C]	Statistical and kinetic parameters			
2.0	144.5				
5.0	154.0	a=	-6.5611		
7.5	159.3	b=	16.0373	k (100°C) [min^{-1}] =	2.94×10^{-3}
10.0	162.7	R^2=	0.9941	k (140°C) [min^{-1}] =	0.124
12.5	164.7	σ=	0.207	k (150°C) [min^{-1}] =	0.281
15.0	169.2	$\sigma_{90\%}$=	0.385		
17.5	170.6	**E_a** [kJ/mol] =	119 ± 7		
20.0	172.5	**Z** [min^{-1}] =	1.57×10^{14}		

REFERENCES

1. Metcalfe, L.D.; Schmitz, A.A.; Pelka, J.R. Rapid Preparation of Fatty Acid Esters from Lipids for Gas Chromatographic Analysis. *Analytical Chemistry* **1966**, *38*, 514-515, doi:10.1021/ac60235a044.
2. Ozawa, T. A New Method of Analyzing Thermogravimetric Data. *Bulletin of the Chemical Society of Japan* **1965**, *38*, 1881-1886, doi:10.1246/bcsj.38.1881.
3. Ozawa, T. Kinetic analysis of derivative curves in thermal analysis. *Journal of Thermal Analysis* **1970**, *2*, 301-324.
4. Wall, L.A.; Straus, S.; Flynn, J.H.; McIntyre, D.; Simha, R. The thermal degradation mechanism of polystyrene. *Journal of Physical Chemistry* **1966**, *70*, 53-62.
5. Litwinienko, G.; Kasprzycka-Guttman, T. Study on the autoxidation kinetics of fat components by differential scanning calorimetry. 2. Unsaturated fatty acids and their esters. *Industrial and Engineering Chemistry Research* **2000**, *39*, 13-17, doi:10.1021/ie990552u.
6. Litwinienko, G. Autooxidation of unsaturated fatty acids and their esters. *Journal of Thermal Analysis and Calorimetry* **2001**, *65*, 639-646, doi:10.1023/A:1017974313294.
7. Musialik, M.; Litwinienko, G. DSC study of linolenic acid autoxidation inhibited by BHT, dehydrozingerone and olivetol. *Journal of Thermal Analysis and Calorimetry* **2007**, *88*, 781-785, doi:10.1007/s10973-006-8507-0.
8. Ostrowska-Ligeza, E.; Bekas, W.; Kowalska, D.; Lobacz, M.; Wroniak, M.; Kowalski, B. Kinetics of commercial olive oil oxidation: Dynamic differential scanning calorimetry and Rancimat studies. *European Journal of Lipid Science and Technology* **2010**, *112*, 268-274, doi:10.1002/ejlt.200900064.
9. Guimarães-Inácio, A.; Francisco, C.R.L.; Rojas, V.M.; Leone, R.d.S.; Valderrama, P.; Bona, E.; Leimann, F.V.; Tanamati, A.A.C.; Gonçalves, O.H. Evaluation of the oxidative stability of chia oil-loaded microparticles by thermal, spectroscopic and chemometric methods. *LWT* **2018**, *87*, 498-506, doi:https://doi.org/10.1016/j.lwt.2017.09.031.
10. Rojas, V.M.; Inácio, A.G.; Martins Fernandes, I.P.; Leimann, F.V.; Gozzo, A.M.; Barros Fuchs, R.H.; Filipe Barreiro, M.F.; Barros, L.; Ferreira, I.C.F.R.; Coelho Tanamati, A.A., et al. Whey protein supplement as a source of microencapsulated PUFA-rich vegetable oils. *Food Bioscience* **2020**, *37*, 100690, doi:https://doi.org/10.1016/j.fbio.2020.100690.
11. Czochara, R.; Ziaja, P.; Piotrowski, P.; Pokrop, R.; Litwinienko, G. Fullerene C 60 as an inhibitor of high temperature lipid oxidation. *Carbon* **2012**, *50*, 3943-3946, doi:10.1016/j.carbon.2012.03.002.
12. Litwinienko, G.; Kasprzycka-Guttman, T. The influence of some chain-breaking antioxidants on thermal-oxidative decomposition of linolenic acid. *Journal of Thermal Analysis and Calorimetry* **1998**, *54*, 203-210, doi:10.1023/A:1010189507864.
13. Ziaja, P.; Jodko-Piorecka, K.; Kuzmicz, R.; Litwinienko, G. Calix[4]pyrogallolarenes as novel high temperature inhibitors of oxidative degradation of polymers. *Polym Chem-Uk* **2012**, *3*, 93-95, doi:10.1039/c1py00494h.
14. Litwinienko, G.; Daniluk, A.; Kasprzycka-Guttman, T. Study on autoxidation kinetics of fats by differential scanning calorimetry. 1. Saturated C12 - C18 fatty acids and their esters. *Industrial and Engineering Chemistry Research* **2000**, *39*, 7-12.
15. Litwinienko, G.; Kasprzycka-Guttman, T.; Studzinski, M. Effects of selected phenol derivatives on the autoxidation of linolenic acid investigated by DSC non-isothermal methods. *Thermochimica Acta* **1997**, *307*, 97-106, doi:10.1016/S0040-6031(97)00366-3.
